# The Student's Manual and Hand-Book for the Laboratory

**Published:** 1890-08

**Authors:** 


THE STUDENT'S MANUAL AND HAND-BOOK FOR THE LABORATORY—Second Edition—By L. P. Haskell, Professor of Prosthetic Dentistry, Dental Department of the North Western University, Chicago. Publishers: Wilmington Dental Manufacturing Company, Philadelphia, 1890. Cloth $1.50. This new edition is larger than the first, and presents a handsome appearance. Although a very useful record of hints, &c., yet it is wanting in minute details and more lengthy descriptions, which its author is well able to present in a much larger work, which would be appreciated by the dental profession.

We hope to see in the near future, a third edition, which will embrace much more matter pertaining to the dental laboratory, than the present edition presents, thus greatly enhancing the value of this manual and rendering it still more useful to the dental student.

